# Dairy Product Consumption Interacts with Glucokinase (GCK) Gene Polymorphisms Associated with Insulin Resistance

**DOI:** 10.3390/jpm7030008

**Published:** 2017-08-30

**Authors:** Marine S. Da Silva, Dominic Chartrand, Marie-Claude Vohl, Olivier Barbier, Iwona Rudkowska

**Affiliations:** 1 Endocrinology and Nephrology, CHU de Québec Research Center and the Department of Kinesiology, Faculty of Medicine, Laval University, Québec, QC G1V 0A6, Canada; marine.da-silva.1@ulaval.ca (M.S.D.S.); dominic.chartrand@criucpq.ulaval.ca (D.C.); 2 Institute of Nutrition and Functional Foods (INAF), and the School of Nutrition, Faculty of Agriculture, Laval University, Québec, QC G1V 0A6, Canada; Marie-Claude.Vohl@fsaa.ulaval.ca; 3 Laboratory of Molecular Pharmacology, CHU de Québec Research Centre and the Faculty of Pharmacy, Laval University, Québec, QC G1V, Canada; olivier.barbier@crchudequebec.ulaval.ca

**Keywords:** dairy, glucose, insulin sensitivity, nutrigenomics, type 2 diabetes

## Abstract

Dairy product intake and a person’s genetic background have been reported to be associated with the risk of type 2 diabetes (T2D). The objective of this study was to examine the interaction between dairy products and genes related to T2D on glucose-insulin homeostasis parameters. A validated food frequency questionnaire, fasting blood samples, and glucokinase (*GCK*) genotypes were analyzed in 210 healthy participants. An interaction between rs1799884 in *GCK* and dairy intake on the homeostasis model assessment of insulin resistance was identified. Secondly, human hepatocellular carcinoma cells (HepG2) were grown in a high-glucose medium and incubated with either 1-dairy proteins: whey, caseins, and a mixture of whey and casein; and 2-four amino acids (AA) or mixtures of AA. The expression of *GCK*-related genes insulin receptor substrate-1 (*IRS-1*) and fatty acid synthase (*FASN*) was increased with whey protein isolate or hydrolysate. Individually, leucine increased *IRS-1* expression, whereas isoleucine and valine decreased *FASN* expression. A branched-chain AA mixture decreased *IRS-1* and *FASN* expression. In conclusion, carriers of the A allele for rs1799884 in the *GCK* gene may benefit from a higher intake of dairy products to maintain optimal insulin sensitivity. Moreover, the results show that whey proteins affect the expression of genes related to glucose metabolism.

## 1. Introduction

The consumption of dairy products has been associated with a lower risk of type 2 diabetes (T2D) [1]. Yet, a recent review of intervention studies by Turner and colleagues [2] demonstrated conflicting results for the effect of dairy product intake on insulin sensitivity: four studies reported improved insulin sensitivity, one reported worsened values, and five reported no effect. Moreover, considerable inter-individual variability has been observed regarding the glucose-insulin response to dairy product consumption, suggesting that such variability may be driven by interactions with genetic factors.

Genome-wide association studies have identified several genetic variations associated with glucose homeostasis (fasting plasma glucose and insulin levels), indirect measures of insulin resistance (such as the homeostasis model assessment of insulin resistance (HOMA-IR)) and the risk of developing T2D [3,4]. Specifically, common genetic variations in the glucokinase (*GCK*) gene are associated with impaired glucose regulation and an increased T2D risk [5,6]. GCK is a key regulator of glucose disposal and storage in both the liver and pancreatic beta-cells. GCK responds to increases in circulating glucose concentrations by initiating a signaling cascade that results in insulin secretion from the beta-cells and subsequent glucose uptake and storage in the liver. In the liver, glucose-sensing enzyme GCK (hexokinase 4) regulates glycogen synthesis and gluconeogenesis, and its activity is competitively inhibited by the glucokinase regulatory protein (GCKR) [7]. Specifically, the insulin-mediated activation of GCK favors glucose utilisation via the activation of key enzymes involved in glycogenosis and lipogenesis pathways, such as insulin receptor substrate-1 (IRS-1) and fatty acid synthase (FASN). If genetic variation reduces the transcription of the *GCK* gene, the cellular activity of GCK would likely decrease and lead to impaired glucose sensing in the liver, an impaired insulin secretion of beta-cells, and eventually diabetes [8]. In diabetic subjects, Haeusler et al. revealed that hepatic *GCK* expression is suppressed by more than 60% compared to non-diabetic subjects [9]. Further, a study reported that nutritional components, such as amino acids, can increase *GCK* expression in liver cells [10].

Clinical studies and reviews have reported that dairy proteins decrease the postprandial glucose response and stimulate insulin secretion, and thus T2D development [11,12]. Cow’s milk contains about 3.5 g of protein per 100 mL, of which whey proteins account for about 20% and casein for 80% [13]. Whey proteins are rich in essential amino acids, specifically branched-chain amino acids (BCAA—leucine, isoleucine and valine). Caseins are characterized by a high proportion of non-essential amino acids such as proline. Consequently, dairy proteins can potentially modify gene expression [14]; therefore, dairy proteins may affect glucose metabolism via the modification of gene expression in key genes regulating glucose homeostasis. 

The objective of the study was to investigate the interaction effects between single-nucleotide polymorphisms (SNPs) within the *GCK* gene and dairy product consumption on variables related to glucose-insulin homeostasis in a cohort of healthy individuals. As dairy proteins may play a role in glucose management, our second objective was to examine in vitro the impact of dairy proteins and amino acids on the expression of key genes related to GCK activation and glucose metabolism in hepatocytes.

## 2. Results

### 2.1. Participants’ Characteristics

Analyses were performed on the 210 participants (97 men and 113 women) who met all the eligibility criteria for the study. Participants aged 30.8 ± 8.7 years old were slightly overweight (body mass index (BMI) 27.9 ± 3.8 kg/m^2^). However, participants had healthy glycemic profiles according to the NCEP-ATP III (National Cholesterol education Program-Adult Treatment Program III) guideline recommendations. Participants consumed an average of 2.4 ± 1.4 portions of dairy products per day. Nearly half (45%) of the individuals did not meet the minimum recommendations for dairy intake from Canada’s Food Guide (two portions of dairy products/day).

### 2.2. Interactions between Glucokinase Genotypes and Dairy Intake

All GCK SNPs were in the Hardy–Weinberg equilibrium (*p* < 0.01), as previously described [15]. We identified a significant interaction between rs1799884 with dairy intake on HOMA-IR (Table 1). 

Specifically, participants who were carriers of the A allele of rs1799884 with lower dairy intake (<2.17 portions/day) had higher HOMA-IR and fasting insulin levels (Table 2). 

### 2.3. Effect of Dairy Proteins and Amino Acids on Glucose Metabolism Genes in HepG2 Cells

Treatments with either dairy proteins or amino acids for 24 h did not affect human hepatocellular carcinoma cells (HepG2) cell viability, which was above 90% for all treatments (data not shown).

**Figure 1:**
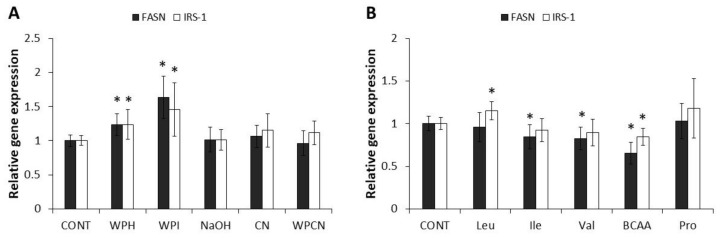
Effect of (**A**) dairy proteins or (**B**) amino acids on mRNA levels of fatty acids synthase (*FASN)* and insulin receptor substrate-1 (*IRS-1)* gene in HepG2 cells. * Significantly different compared to control (*p* < 0.05). CONT: control; WPH: hydrolysate of whey proteins; WPI: isolate of whey proteins; CN: caseins; NaOH: sodium hydroxide; WPCN: mixture of caseins and whey proteins; BCAA: branched-chain amino acids; Leu: leucine; Ile: isoleucine; Val: valine; Pro: proline.

*FASN* and *IRS-1* gene expression was determined following treatments with dairy proteins for 24 h (Figure 1a). Both the hydrolysate (WPH) and isolate (WPI) of whey proteins increased *FASN* and *IRS-1* gene expression. WPH increased *FASN* and *IRS-1* by 1.24-fold (*p* = 0.004) and 1.24 fold (*p* = 0.02), respectively, whereas WPI raised *FASN* and *IRS-1* by 1.63 fold (*p* = 0.02) and 1.46 fold (*p* = 0.03), respectively. Caseins (CN) and the mixture of caseins and whey proteins (WPCN) did not affect *FASN* and *IRS-1* gene expression. 

The effect of amino acids on *FASN* and *IRS-1* gene expression is shown in Figure 1b. A mixture of BCAA decreased *FASN* gene expression (fold expression: 0.65, *p* < 0.0001). Consistently, individual BCAAs isoleucine (fold expression: 0.85, *p* = 0.02) and valine (fold expression: 0.83, *p* = 0.005), but not leucine, also decreased *FASN* gene expression. While BCAA decreased *IRS-1* gene expression (fold expression: 0.85, *p* = 0.002), isoleucine and valine had no effect. Moreover, leucine increased *IRS-1* gene expression (fold expression: 1.15, *p* = 0.004). Proline affects neither *FASN* nor *IRS-1* gene expression. 

### 2.4. Glucose Concentrations in Cell Supernatants are Linked to FASN and IRS-1 Gene Expression

**Figure 2:**
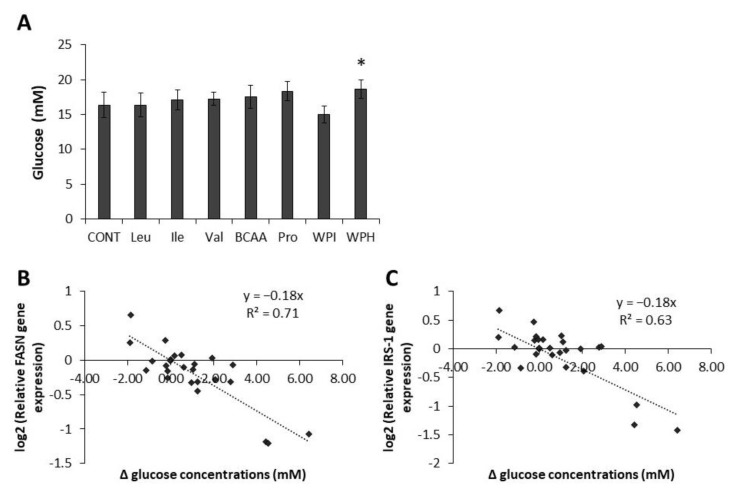
Effect of dairy proteins and amino acids on glucose concentration in cell supernatants. (**A**) Glucose concentration in cell supernatants following treatments with WPH or WPI (5 mg/mL), or amino acids (20 mM) for 24 h.* Significantly different compared to control (*p* < 0.05); (**B**) and (**C**) Regression analysis between glucose concentrations in supernatants and *FASN* or *IRS-1* gene expression. Δ glucose concentrations = [Treatment] − [Control].

Glucose concentrations in supernatants following treatments with whey proteins and amino acids are shown in Figure 2a. Overall, glucose concentrations did not appear to be affected by dairy protein or amino acid treatments, except for the WPH treatment, in which the glucose concentration was increased compared to the control (*p* = 0.03). Yet, the regression analysis revealed that glucose concentrations in cell supernatants were linked to both *FASN* (R^2^ = 0.71, *p* < 0.0001) and *IRS-1* (R^2^ = 0.63, *p* < 0.0001) gene expression (Figure 2b,c). Specifically, there was a linear relation between the difference in glucose concentrations and both *FASN* and *IRS-1* gene expression with a slope around −0.2 for both genes. This means that when *FASN* or *IRS-1* gene expression is increased, the glucose concentrations in the corresponding supernatant are lower than the control treatment. 

## 3. Discussion

The results suggest that the intake of dairy products may be associated with glucose-insulin homeostasis in individuals with specific SNPs related to the risk of T2D. This study showed that the *GCK* SNP rs1799884 interacts with dairy product consumption and is associated with glucose-insulin homeostasis parameters. Further, our results indicate that dairy proteins, specifically whey proteins, may improve glucose metabolism by increasing glucose utilisation in the liver.

Participants with a low intake of dairy products and the promoter variant (−30A) rs1799884 in *GCK* have higher HOMA-IR levels. Rare mutations in the *GCK* gene have been found to be associated with maturity-onset diabetes of the young (MODY), permanent neonatal diabetes, and hyperinsulinemia of infancy [16]. The rs1799884 variant in *GCK* has been found to be associated with an increased risk of diabetes, hyperglycaemia, and impaired beta-cell function [6,17,18,19,20]. Mice that are lacking hepatic GK suffer from impaired insulin secretion [21]. By contrast, the hepatic overexpression of GCK in diabetic or non-diabetic mice resulted in improved glucose tolerance [21]. Therefore, genetic variations in *GCK* that lower *GCK* expression may impact GCK enzyme activity, which could increase susceptibility for T2D. A recent study calculated the genetic risk score on the basis of 31 diabetes-associated variants, including a SNP in *GCK* (rs4607517) [22]. This study suggests that a high-protein diet may be more beneficial for individuals with a higher genetic risk to improve insulin resistance and beta-cell function [22]. Similarly, these results demonstrate that individuals with SNPs in *GCK* may benefit from a higher intake of dairy products.

Our in vitro study demonstrated that dairy proteins may have beneficial effects on insulin sensitivity by increasing glucose utilisation in the liver. In this study, we used two forms of whey proteins: 1-WPI contains 85–90% protein and very little fat or lactose and 2-WPH consists of proteins that have undergone hydrolysis by proteolytic enzymes [13]. Specifically, in the WPH, the proteins have been broken down into smaller, more easily digestible peptide and amino acid components. This study demonstrated that WPI and WPH increased the gene expression of both *IRS-1* and *FASN*, while caseins and the mixture of caseins and whey proteins had no effect. Consequently, one form of dairy protein may be more functional than another to improve glucose metabolism. In addition, the beneficial outcome of whey proteins may be in part due to their amino acid profile.

Whey protein is a rich source of BCAA leucine, isoleucine, and valine, which have the potential to improve glucose metabolism [23]. Contrary to our results with whey proteins, the BCAA mixture decreased the expression of *FASN* and *IRS-1* in HepG2 cultivated in a high glucose (25 mM) medium. Similarly, a research group showed that the addition of a mixture of BCAA did not affect the expression levels of glucose transporter 2 (*GLUT2*) in hepatocytes cultivated in low glucose conditions (5.5 mM) [10]. Yet, the same study demonstrated that BCAA strongly increased the expression of *GLUT2* and liver-type glucokinase (*L-GK*) in high glucose (22 mM) conditions. These results suggest that the presence of BCAA in high glucose conditions may be necessary to promote the maximal expression of the glucose-sensing apparatus in HepG2 cells. These opposing results may be due to the ratio of amino acids in the BCAA mixture (2:1:1.2, leucine/isoleucine/valine) that was dissimilar (1:1:1, leucine/isoleucine/valine). Accordingly, individual amino acids were examined.

Leucine, the most abundant BCAA in dairy proteins, especially whey proteins, increased *IRS-1* gene expression. This is in accordance with our results with whey proteins but not our mixture of BCAA. However, the ratio of leucine: isoleucine: valine in the BCAA mixture was equimolar compared to the study by Higuchi et al. [10], where leucine in the BCAA mixture was twice the concentration of isoleucine or valine. In accordance with the mixture of BCAA, isoleucine and valine decreased *FASN* expression. Thus, the beneficial effect of leucine may have been overshadowed by the negative effects of isoleucine and valine in the BCAA mixture. In addition, proline affects neither *FASN* nor *IRS-1* gene expression. The proportion of proline is higher in caseins than in whey proteins; thus, in agreement with our results with caseins. Taken together, this showed that amino acids can mimic some, but not all, of the effects of dairy proteins. Further, more in-depth mechanistic studies are needed to better understand the bioactive components in whey proteins.

First, the sample size is relatively small; thus, these results need to be validated in larger well-characterised cohorts, but should be tested for validity in a clinical trial with biomarkers of diabetes. Second, there may be unobserved differences in participants’ characteristics between the high dairy and low-dairy groups. Third, the intake of dairy products is heterogeneous; therefore, further studies are needed to be able to better characterise dairy intake. Finally, this study tested one genetic variant for diabetes when more than 40 genetic risk variants for T2D have been validated. More sophisticated studies for testing gene-diet interaction effects should be developed to test the effects of multiple SNPs, such as the genetic risk score. 

## 4. Materials and Methods

### 4.1. Human Study

#### 4.1.1. Study Population

A total of 254 of healthy participants from the greater Quebec City metropolitan area were recruited. Study inclusion and exclusion criteria’s have been previously described [24]. The following baseline values of the clinical trial were collected from each of the study participants: anthropometric measurements, fasting blood samples, and a food frequency questionnaire (FFQ). Results were obtained from 210 participants. Specifically, a group size per group (*n* = 52) was necessary to provide an 80% probability at *p* < 0.05 of detecting an anticipated difference of in insulin resistance (−0.18 ± 0.10), as calculated with the HOMA-IR. In addition, this sample size will allow us to determine if genetic variability affects insulin resistance in wild-type compared to minor allele carrier (MAF > 10%). In previously published studies from our research group, this sample size was deemed appropriate to investigate gene-diet interactions [15,25]. The protocol was approved by the ethics committees of Laval University Hospital Research Center. This trial was registered at clinicaltrials.gov as NCT01343342.

#### 4.1.2. Anthropometric Measurements

Body weight, height, waist, and hip circumferences were measured according to the procedures recommended by the Airlie Conference [26]. BMI was calculated as weight in kilograms divided by height in meters squared. Measurements were performed in duplicate and the mean was used for analyses.

#### 4.1.3. Dietary Intake

Dietary intake of the past month was determined by a 91-item validated FFQ [27] based on the food habits of Quebecers, administered by a registered dietitian (RD). The RD asked participants how often they consumed each type of food: daily, weekly, monthly, or none at all during the last month. We defined a dairy portion/day by 250 mL of milk, 175 g of yogurt or frozen yogurt, 50 g of cheese, or 250 mL of cottage cheese. To make sure that each participant correctly estimated the portion eaten, examples of portion sizes were provided. Data obtained from FFQ were analysed using the Nutrition Data System for Research software version 2011, developed by the Nutrition Coordination Center (University of Minnesota, Minneapolis, MN, USA). 

#### 4.1.4. Biochemical Parameters

Blood samples were collected from an antecubital vein into vacutainer tubes containing EDTA after a 12-h overnight fast and 48-h alcohol abstinence. Plasma was separated by centrifugation (2500× *g* for 10 min at 4 °C) and samples were aliquoted and frozen at −80 °C for subsequent analyses. Fasting insulin was measured by a radioimmunoassay with polyethylene glycol separation [28]. Fasting glucose concentration was measured enzymatically [29]. Insulin resistance was calculated using the HOMA-IR: HOMA-IR = fasting insulin (IU/mL) × fasting blood glucose (mmol/L)/22.5 [30]. One HOMA-IR value of more than 10 (the mean plus/minus a coefficient three times the standard deviation [31]) was excluded from the statistical analysis and three values were missing.

#### 4.1.5. DNA Extraction and Genotyping 

The SIGMA GenElute Gel Extraction Kit (Sigma-Aldrich Co., St. Louis, MI, USA) was used to extract genomic DNA. Selected SNPs were genotyped using validated primers and TaqMan probes (Applied Biosystems, Foster City, CA, USA). DNA was mixed with TaqMan Universal PCR Master Mix (Applied Biosystems) and a gene-specific primer and probe mixture (predeveloped TaqMan SNP Genotyping Assays; Applied Biosystems) in a final volume of 10 μL. Genotypes were determined using a 7500 FAST real time-PCR (RT-PCR) System and analyzed using ABI Prism SDS version 2.0.5 software (Applied Biosystems).

As described previously [15], SNPs in GCK were identified using the International HapMap Project SNP database, based on the National Center for Biotechnology Information (NCBI) B36 assembly Data Rel 28, phase II + III, build 126. The tagger procedure in Haploview V4.2 was used to determine tag SNPs (tSNPs) using a minor allele frequency (MAF) > 5% and pairwise tagging (r^2^ ≥ 0.8). Overall, thirteen tSNPs, covering 86% of the known genetic variability within the GCK gene (including 2500 bp upstream and 500 bp downstream GCK gene), were genotyped: rs2268573; rs2908297; rs2971676; rs758989; rs12673242; rs2908290; rs2284777; rs2300584; rs1990458; rs741038; rs1799884; rs2908277; rs3757838.

### 4.2. In Vitro Study

In vitro experiments were carried out to examine the modulation of GCK-related genes by dairy proteins.

#### 4.2.1. Materials

Non-hydrolysed WPI 90 (WPI) was from Milk Specialties Global (Fond du Lac, WI, USA) and Lacprodan^®^ DI-3095 WPH was from Arla Food Ingredients (Viby J, Denmark). Minimum protein content of WPI and WPH was, respectively, 90% and 82%. Casein from bovine milk (minimum protein content 87%) was purchased from Sigma-Aldrich. l-leucine, l-isoleucine, l-valine, and l-proline were purchased from MP Biomedicals LLC (Solon, OH, USA). The TRI Reagent was supplied by Molecular Research Center Inc. (Cincinnati, OH, USA). The High Capacity cDNA Archive Kit, TaqMan gene expression assays, and Taqman Fast Advanced Master Mix were obtained from Applied Biosystems.

#### 4.2.2. Cell Culture and Treatment

HepG2 were grown in high glucose conditions (25 mM) in Dulbecco’s Modified Eagle’s Medium (DMEM) supplemented with 10% fetal bovine serum (FBS), 1% of streptomycin penicillin, 1% of sodium pyruvate, and 1% of glutamine at 37 °C in 5% CO_2_ in a humidified atmosphere. HepG2 cells were grown until 70% cell confluence was reached, and were then seeded at a density of 3.0 × 10^5^ cells mL^−1^ in 12-well plates for 16 h. On the day of the experiments, solutions of WPI (5 mg/mL), WPH (5 mg/mL), leucine (20 mM), isoleucine (20 mM), valine (20 mM), proline (20 mM), or a mixture of leucine, isoleucine, and valine (BCAA, 20/20/20 mM) were prepared in serum-free DMEM containing 25 mM of glucose and were added to the cells. These physiological concentrations were chosen according to previous reports [32], the plasma amino acid values after the ingestion of dairy proteins [33], and solubility. Serum-free DMEM containing 25 mM of glucose was used as the control. Sodium hydroxide (NaOH, 1 M) was used to prepare the casein stock solution (50 mg/mL). This solution was then diluted in serum-free DMEM containing 25 mM of glucose to prepare the casein (1 mg/mL) and the mixture of WPI and casein (WPCN, 0.25/1 mg/mL) treatments. For WPCN, the ratio 1:4 (*w*/*w*) was selected to represent the proportions of whey proteins and caseins in milk. The control for the casein and WPCN treatments was serum-free DMEM containing 25 mM of glucose and the same quantity of NaOH (0.02 M). Each experiment was carried out in triplicate. Following the 24-h treatments, cell supernatants were collected and kept at −80 °C for the determination of glucose concentrations, while RNA was collected for the gene expression analysis.

#### 4.2.3. Gene Expression Analysis

Total RNA was isolated from control or treated cells according to the TRI Reagent acid: phenol protocol, as specified by the supplier. After the spectrophotometric quantification and verification of the total RNA quality, complementary NDA (cDNA) was generated from 1 μg of total RNA and stored in aliquots at −20 °C until the analyses. Gene expression of *IRS-1* (Hs00178563_m1) and *FASN* (Hs01005622_m1) was determined by quantitative PCR, using TaqMan gene expression assays and the Viia7 Real-Time PCR System instrument from Applied Biosystems. For each reaction, the final volume of 10 μL was comprised of 5 μL of Taqman Fast Advanced Master Mix, 0.5 μL of TaqMan Gene Expression assay, and 0.5 μL of cDNA. Conditions for RT-PCR were 95 °C for 20 s, 95 °C for 1 s, and 60 °C for 20 s for 40 cycles. Threshold cycle (Ct) values were analysed using the comparative Ct (ΔΔCt) method [34]. The amount of target gene (2^−ΔΔCt^), normalized to β-actin gene expression (Hs99999903_m1), is represented as the fold change in mRNA expression relative to the appropriate control, set at 1.

#### 4.2.4. Glucose Concentrations in Cell Supernatants

Glucose concentrations in the supernatants of treated cells were measured using the Bayer Contour Next EZ Glucose Meter and test strips (Bayer HealthCare LLC, Mishawaka, IN, USA). 

#### 4.2.5. Cell Viability Assay

A CellTiter96 Aqueous Cell Proliferation assay from Promega (Madison, WI, USA) was used to test the viability of HepG2. HepG2 cells were seeded in a 96-well plate at a density of 20,000 cells/well and treated as described. Then, 20 μL (3-(4,5-dimethylthiazol-2-yl)-5-(3-carboxymethoxyphenyl)-2-(4-sulfophenyl)-2H-tetrazolium inner salt (MTS)/phenazine methosulfate (PMS—electron coupling reagent) per well were added and cells were incubated 2 h at 37 °C in 5% CO_2_. The optical density of the MTS bioreduction product formazan was measured at 490 nm with the TECAN plate reader (TECAN, Männedorf, Switzerland).

### 4.3. Statistical Analyses 

For the human study, data are shown as mean ± standard error (SE) unless otherwise stated. The Hardy-Weinberg equilibrium (HWE) was tested with the Allele Procedure in SAS (SAS Institute Inc., Cary, NC, USA). For some SNPs, heterozygotes and minor allele homozygotes were grouped if the genotype frequency was under 5%. All data were checked for skewness and kurtosis and normalized by log transformation when necessary (insulin and HOMA-IR). Dairy product intake was evaluated as portions per day, and then dichotomized into high- and low-intake based on the population median. The Analysis of Variance Model (ANOVA) was used to test for the effects of the *GCK* genotypes, dairy intake, and the genotypes by dairy intake interaction on glycemic parameters (fasting insulin; fasting glucose; HOMA-IR). Since it is well established that glycemic parameters behave differently according to age, gender, and obesity status, the analyses were adjusted for age, sex, and BMI [35]. Polymorphisms tested in complex diseases rarely account for a large proportion of the variance, and therefore, the results are presented before correction for multiple testing and using a *p* ≤ 0.05 [25].

For the in vitro study, all data are presented as the mean ± standard deviation (SD) of three separated experiments. Two-tailed unpaired Student *t*-tests were used to assess the difference between one treatment and the control, with significance accepted at values of *p* ≤ 0.05. The relation between glucose levels in supernatants and the gene expression of *IRS-1* and *FASN* was analyzed by linear regression. The difference in glucose concentrations between each dairy proteins/amino acid treatments and the control treatment was defined as the dependent variable, whereas either *IRS-1* or *FASN* gene fold expression was the independent variable.

Data were analyzed with SAS statistical software, version 9.4 (SAS Institute Inc.).

## 5. Conclusions

The results indicate that the intake of dairy products, specifically whey proteins, may be associated with glucose-insulin homeostasis in individuals with SNPs related to the risk of diabetes. Specifically, our in vitro hepatic model shows that whey proteins modify genes related to glucose metabolism. This study provides new hypotheses to incorporate dairy products for specific the group of people at risk of T2D; thus, it may help to devise more personalized nutrition in preventing T2D. Additional studies in larger cohorts and clinical trials are needed.

## Figures and Tables

**Table 1:** Gene-diet interaction effects for total dairy intake on homeostasis model assessment of insulin resistance (HOMA-IR) according to tag single nucleotide polymorphism (tSNP) in glucokinase (*GCK)*.

tSNP	Localisation	Genotype (*n*)	*p*-Values ^1^		
			Genotype	Dairy	Interaction
rs2268573 C > A MAF = 0.39	Intron	CC (39) AC (111) AA (60)	0.93	0.91	0.24
rs2908297 G > A MAF = 0.31	Intron	GG (167) AG (36) + AA (7)	0.94	0.99	0.96
rs2971676 C > T MAF = 0.10	Intron	CC (183) CT (24) + TT (3)	0.94	0.68	0.60
rs758989 A > G MAF = 0.37	Intron	AA (62) AG (96) GG (52)	0.62	0.90	0.10
rs12673242 T > C MAF = 0.24	Intron	TT (171) CT (36) + CC (3)	0.27	0.59	0.53
rs2908290 C > T MAF = 0.45	Intron	CC (102) CT (88) TT (20)	0.84	0.43	0.34
rs2284777 G > A MAF = 0.12	Intron	AA (152) AG (50) + GG (8)	0.89	0.70	0.30
rs2300584 T > C MAF = 0.19	Intron	TT (134) CT (70) + CC (6)	0.97	0.75	0.21
rs1990458 G > A MAF = 0.34	Intron	GG (79) AG (100) AA (31)	0.59	0.69	0.69
rs741038 C > T MAF = 0.28	Intron	TT (93) CT (94) CC (23)	0.97	0.38	0.08
rs1799884 G > A MAF = 0.17	5′UTR	GG (135) AG (65) + AA (10)	0.93	0.54	0.02
rs2908277 C > T MAF = 0.20	3′UTR	CC (166) CT (41) + TT (3)	0.78	0.80	0.67
rs3757838 A > T MAF = 0.05	Promoter	TT (186) AT (21) + AA (3)	0.53	0.59	0.49

^1^ The model includes the effects of the genotypes, the dairy intake (low versus high intake, according to the median intake of 2.17 portions/day), and the genotype by dairy intake interaction effect on HOMA-IR, adjusted for age, sex, and body mass index (BMI). MAF: minor allele frequency.

**Table 2:** Effect of the interaction between rs1799884 and dairy intake (median: 2.17 portions/day) on glucose-related parameters.

Genotype	GG (*n* = 135)		AG (*n* = 65) + AA (*n* = 10)		*p*-Values		
Dairy intake	Low (*n* = 66)	High (*n* = 68)	Low (*n* = 38)	High (*n* = 37)	G	D	G × D
Fasting glucose	4.98 ± 0.42	4.89 ± 0.48	5.11 ± 0.46	4.85 ± 0.45	0.30	0.21	0.33
Fasting insulin	79.25 ± 37.44	84.91 ± 35.58	88.24 ± 49.75	70.78 ± 27.76	0.66	0.89	0.035
HOMA-IR	2.54 ± 1.27	2.65 ± 1.09	2.88 ± 1.60	2.21 ± 0.88	0.93	0.54	0.02

The model includes the effects of the genotype (G), the dairy intake (D) and the genotype by dairy intake interaction (G × D) on HOMA-IR, adjusted for age, sex and BMI.
